# Kaumoebavirus, a New Virus That Clusters with Faustoviruses and *Asfarviridae*

**DOI:** 10.3390/v8110278

**Published:** 2016-10-28

**Authors:** Leena H. Bajrai, Samia Benamar, Esam I. Azhar, Catherine Robert, Anthony Levasseur, Didier Raoult, Bernard La Scola

**Affiliations:** 1 Unité des Rickettsies, URMITE UMR CNRS 7278 IRD 198 INSERM U1095, Facultés de Médecine et de Pharmacie, IHU Méditerranée Infection, Aix-Marseille Université, Marseille 13005, France; leenaasd@gmail.com (L.H.B.); benamarsamia@yahoo.com (S.B.); catherine.robert@univ-amu.fr (C.R.); anthony.levasseur@univ-amu.fr (A.L.); didier.raoult@gmail.com (D.R.); 2 Department of Biochemistry, Faculty of Science, King Abdulaziz University, 21362 Jeddah, Saudi Arabia; 3 Special Infectious Agents Unit, King Fahd Medical Research Center, and Department of Medical Laboratory Technology, Faculty of Applied Medical Sciences, King Abdulaziz University, 21362 Jeddah, Saudi Arabia; eazhar@kau.edu.sa

**Keywords:** Kaumoebavirus, *Vermamoeba vermiformis*, Faustoviruses, Asfarviruses

## Abstract

In this study, we report the isolation of a new giant virus found in sewage water from the southern area of Jeddah (Saudi Arabia), with morphological and genomic resemblance to Faustoviruses. This new giant virus, named Kaumoebavirus, was obtained from co-culture with *Vermamoeba vermiformis*, an amoeboid protozoa considered to be of special interest to human health and the environment. This new virus has ~250 nm icosahedral capsids and a 350,731 bp DNA genome length. The genome of Kaumoebavirus has a coding density of 86%, corresponding to 465 genes. Most of these genes (59%) are closely related to genes from members of the proposed order *Megavirales*, and the best matches to its proteins with other members of the *Megavirales* are Faustoviruses (43%) and Asfarviruses (23%). Unsurprisingly, phylogenetic reconstruction places Kaumoebavirus as a distant relative of Faustoviruses and Asfarviruses.

## 1. Introduction

The story of giant viruses began with the discovery of Mimivirus [1,2], the first giant virus to be described and which has now been isolated from many environmental and human samples [3,4,5,6,7,8,9]. Subsequently, other viruses such as Marseilleviruses [4,10,11,12], *Cafeteria roenbergensis* virus (CroV) [5], Pandoraviruses [13], *Pithovirus sibericum* [14] and *Mollivirus sibericum* [15] were discovered using *Acanthamoeba* sp. as a support for co-culture, with the exception of CroV, which was co-isolated alongside its host, the marine flagellate *Cafeteria roenbergensis*. These viruses were recognized as members of the nucleocytoplasmic large DNA viruses (NCLDVs) clade, and it was recently proposed that they should be merged into *Megavirales*, a new viral order [16]. In 2015, Faustovirus, a new Asfarvirus lineage of giant viruses, was discovered [17]. It was the first to be isolated by co-culture with an amoeba other than *Acanthamoeba* sp., *Vermamoeba vermiformis*, and later additional isolates were obtained [18]. Using the same support for co-culture, in this paper, we report the isolation and genome analysis of a new virus we named Kaumoebavirus (meaning King Abdulaziz University amoeba virus), isolated from an environmental sample (sewage water) obtained in the southern area of Jeddah, Saudi Arabia. 

## 2. Materials and Methods

### 2.1. Isolation and Routine Subculture

A sewage water sample collected from the southern area of Jeddah was inoculated onto a *V. vermiformis* (strain CDC19) monolayer in starvation medium at 30 °C, as previously described in detail [19]. Viral particles observed in the culture supernatant were serially diluted from 10^−1^ to 10^−11^ using the end-point dilution method. Briefly, microplates were inoculated with each dilution in four wells that already contained rinsed *V*. *vermiformis* at 10^6^ per mL. Only one well that simultaneously had a complete lysis at 30 °C in the highest dilution was used for virus production [20]. Viral particles were purified as previously described [19]. 

### 2.2. Studying the Viral Cycle Using TEM

A co-culture was prepared by inoculating a 25 cm^2^ cell culture flask in *V. vermiformis* starvation medium at 10^6^ cells per mL [17] with Kaumoebavirus at 30 °C at a ratio of 1:10. Thirty minutes post-infection (PI), the *V. vermiformis*-infected monolayer was recovered and pelleted for 10 min at 5000× *g*, which was considered as 0 h. The pellet was suspended in 1 mL of phosphate buffered saline (PBS), fixed in 1 mL of 2% glutaraldehyde 0.1 M cacodylate buffer, and then incubated for at least 1 h at 4 °C, preparing for transmission electron microscopy (TEM). Then, 200 µL infected cells were cytocentrifugated on two slides for Hemacolor and DAPI for nucleic acid labeling (Molecular Probes, Oregon, USA). This step was repeated every 4 h for the next 28 h PI. All samples were prepared for electron microscopy as previously described [17]. Grids were observed under Tecnai G2 transmission electron microscope (FEI, Oregon, USA) at 200 keV through ultrathin 70 nm sections. 

### 2.3. Genome Study

The Kaumoebavirus genome was sequenced as previously described [17] by MiSeq Technology (Illumina, Inc., San Diego, CA, USA) using paired-end and mate-pair applications in parallel, in a 2× 251 bp run for each bar-coded library. The reads were assembled de novo into a contig using the Spades assembler software v1.0 combined with GapFiller, which was used to enhance the assembly [21,22]. Protein-coding regions were predicted using GeneMark [23]. tRNA were searched using ARAGORN software [24].

The phylogenetic trees were built by protein sequence alignment using the MUSCLE program with the default parameters [25]. Phylogeny reconstructions were performed for Kaumoebavirus and other *Megavirales* members by the maximum likelihood method using FastTree [26], with the default parameters (JTT evolutionary model, discrete gamma model with 20 rate categories), based on the conserved genes. 

A comparative analysis of the Kaumoebavirus genome and those of the *Asfarviridae* and Faustoviruses genomes was generated by creating a reference database of all the protein sequences. COGtriangles [27] and OrthoMCL [28] clustering algorithms were used to create protein clusters. Pangenome and core genes were defined using GET_HOMOLOGUES [29] with the following parameters: 75% as minimum coverage, 30% as minimum identity in BLASTP pairwise alignments and 1 × 10^−5^ as maximum E-value.

### 2.4. Accession Number

The complete genome sequence of Kaumoebavirus was submitted to GenBank and assigned the accession number KX552040.

## 3. Results

### 3.1. Replication Cycle

**Figure 1:**
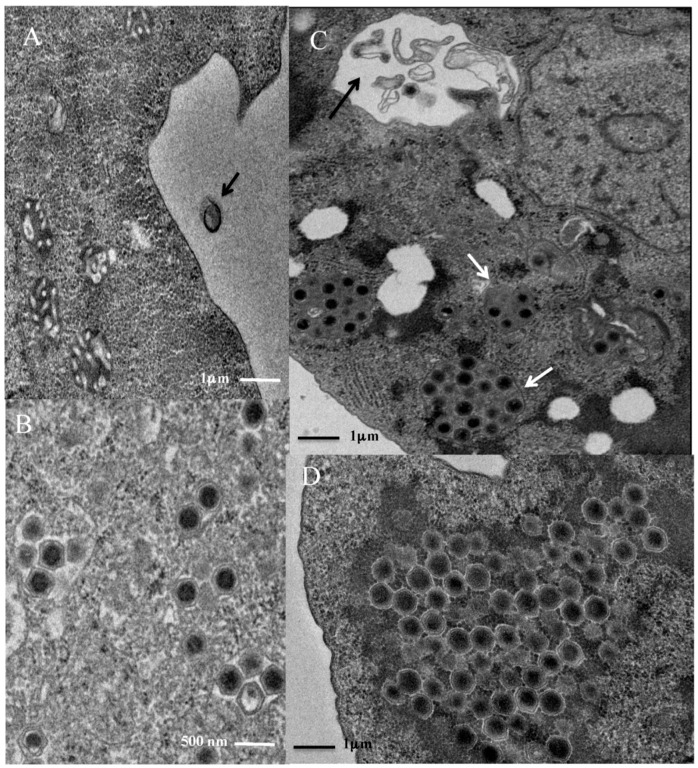
Observation of Kaumoebavirus in *Vermamoeba vermiformis* at selected times of development. Kaumoebavirus particles (black arrow) are phagocytized (**A**) then observed in the cytoplasm of *V. vermiformis* mostly packaged in clumps of two to four particles (**B**). After the eclipse phase, new virions may be observed within the virus factory (**C**,**D**). During the early stage of its formation, the virus factory of Kaumoebavirus is not round-shaped but poly-lobed. During the microtome cut, some slice planes showed newly formed viruses in these lobes as clusters of virions (white arrows). At this stage, remnants of phagocytized particles may be observed (black arrow) (**C**). At a late stage, the virus factory loses its poly-lobed aspect for a large round vacuole aspect and appears completely filled with Kaumoebavirus mature particles (**D**).

At an early stage (two hours PI), we observed the entry of Kaumoebavirus particles through phagocytosis into the *V. vermiformis* cytoplasm (Figure 1A). After phagocytosis, at three hours PI, the viral particles were mostly located as clumps of two to four particles within vacuoles (Figure 1B). At four hours PI, the “eclipse” phase was obvious, as no viruses could be observed within the cytoplasm or only few visible vacuoles appeared empty, which is likely due to the release of the viral DNA. Virus factories could be observed in the cytoplasm and close to the nucleus at six hours PI. Newly-formed mature virions of Kaumoebavirus were observed from eight to 16 h PI accumulating within the virus factory (Figure 1C). The nuclear morphology of the host cell did not change during the Kaumoebavirus replication cycle. During the final stages of morphogenesis (20 h PI), viral particles accumulated massively within the virus factory (Figure 1D) before being released from each infected cell via lysis. 

### 3.2. Genomic Features

**Figure 2:**
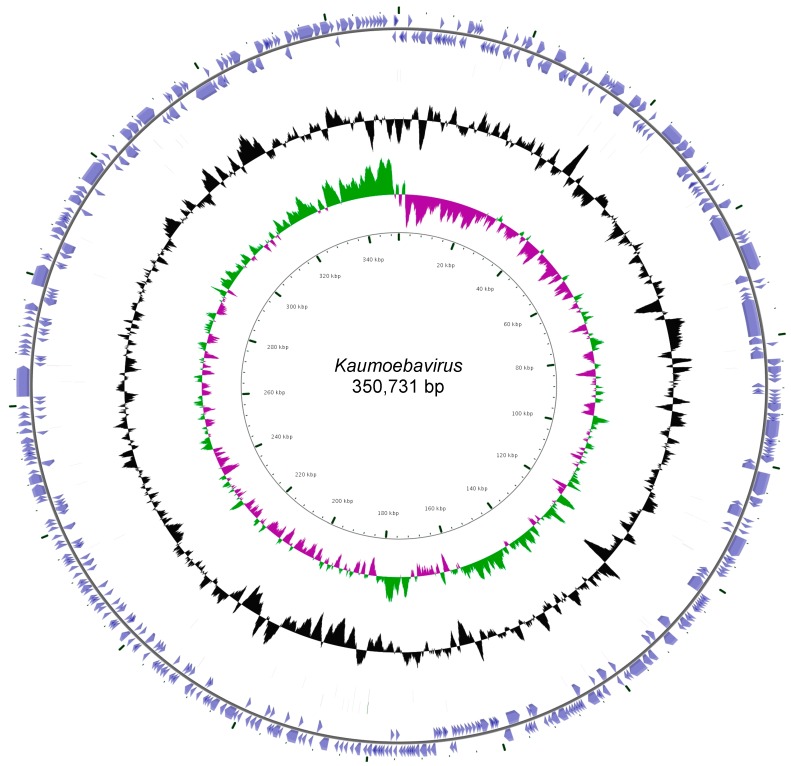
Circular representation of the Kaumoebavirus genome. The circles from the center to the outside show: GC skew (green/purple); GC content (black); open reading frames (ORFs) on the plus and minus strands (blue).

The Kaumoebavirus genome is a circular double stranded DNA (dsDNA) of 350,731 bp (Figure 2). The genome is devoid of internal repeats (Figure S1), except for a unique long inverted tandem repeat of 1407 bp length. 

The Kaumoebavirus genome has a global GC content of 43.7% and a GC value of 44.5% when only coding sequences are considered. The GC content value of genes ranging from 28.8% to 60%, standard deviations (3.93) and quartiles (first quartile: 41.6, third quartile: 46.2) were calculated. Values exceeding the third quartile were classified as GC-rich sequences, while those inferior to the first quartile formed GC-poor sequences. Half of the Kaumoebavirus genes were classified as GC-poor (117) or GC-rich (118). More than 70% of these genes have no match against the non-redundant (NR) database. A relationship between GC content and gene length could be observed. The GC-rich genes are mainly the longest coding sequences (mean: 872 bp, first quartile: 306 bp, third quartile: 1089 bp), while the GC-poor genes are the shortest (mean: 326 bp, first quartile: 189 bp, third quartile: 580 bp).

**Figure 3:**
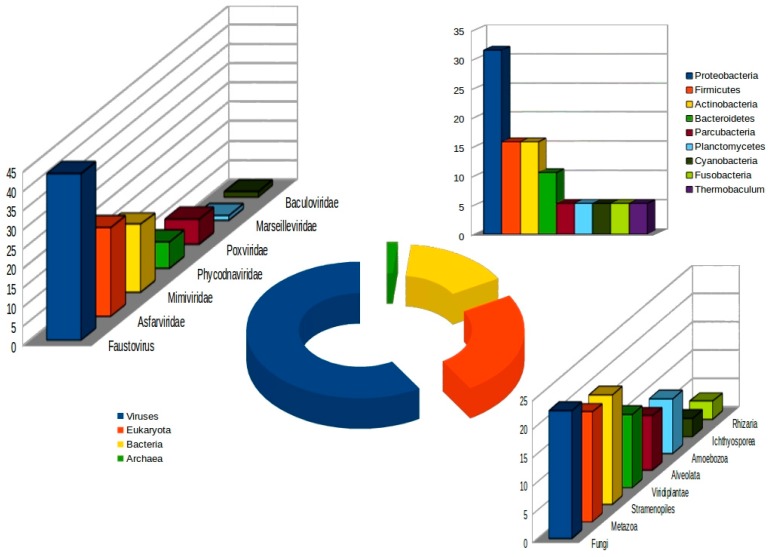
Distribution of the best-matching homologs to Kaumoebavirus proteins (the diagram shows only the 59% of genes with significant BLAST hits). Best-matching homologous proteins were determined using BLASTP (E value < 10^−3^) against the non-redundant (NR) database at the National Center for Biotechnology Information (NCBI).

We identified at total number of 465 proteins (amino acid numbers ranged from 113 aa to 6209 aa), representing an 86% coding density. ARAGORN software did not predict tRNA genes. The sequences were analyzed using BLAST (with an E-value threshold of 10^−2^) against the non-redundant protein sequence database. Of the Kaumoebavirus proteins which had homology in the databases, 74 (59%) were most similar to a virus protein, 19 (15%) to bacteria proteins, 31 (25%) to proteins of other eukaryotes, and two (2%) to Archaea proteins (Figure 3). 

**Figure 4:**
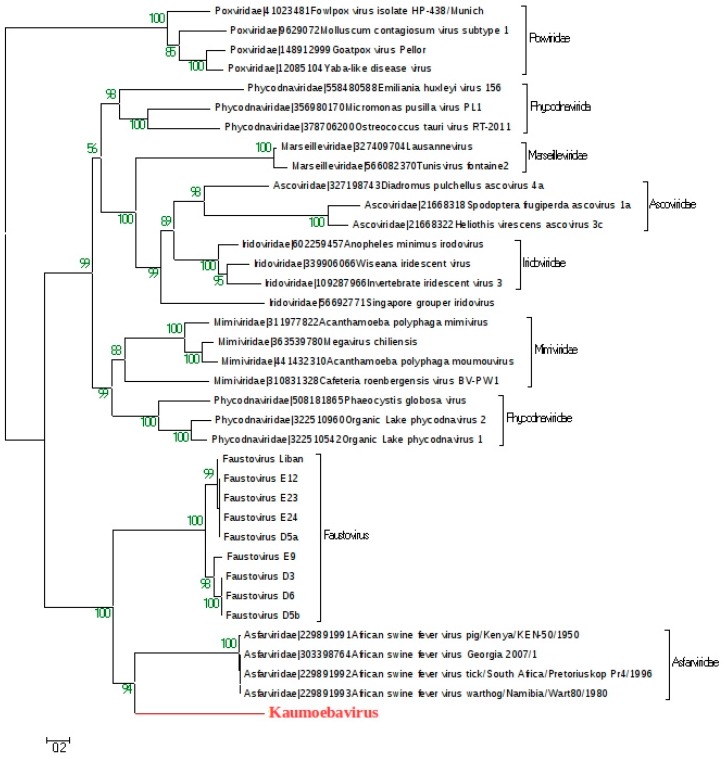
Phylogenetic reconstruction based on a concatenated A32-like packaging ATPase and the family B DNA polymerase. Phylogenetic analysis was performed using the maximum likelihood method based on protein sequences from Kaumoebavirus and representative members from the different families of *Megavirales* order.

For 59% of the Kaumoebavirus proteins with detectable homologs to virus proteins, the best matches were to proteins from other members of the *Megavirales*, mostly Faustoviruses (32 cases; 43%) and Asfarviruses (17 cases; 23%). The remaining best viral matches were to proteins from Mimiviruses, Phycodnaviruses, *Poxviridae*, Marseilleviruses and *Baculoviridae* in 13, 5, 5, 1 and 1 cases, respectively. Despite the fact that Kaumoebavirus has a significant number of homologs to *Megavirales* proteins, no detectable collinearity between the Kaumoebavirus and Faustovirus E9, Faustovirus Liban, or African swine fever virus E75 could be observed. This suggests that the basic structure of the Kaumoebavirus genome is original, despite the significant number of shared proteins (Supplementary Materials Figure S2). However, as compared with other *Megavirales* members, the gene content of Kaumoebavirus is globally more similar to those of Faustoviruses and *Asfarviridae* than other *Megavirales*. This observation is illustrated by the phylogenetic clustering of the concatenated Kaumoebavirus protein sequences of the A32-like packaging ATPase and the family B DNA polymerase (Figure 4). 

**Figure 5:**
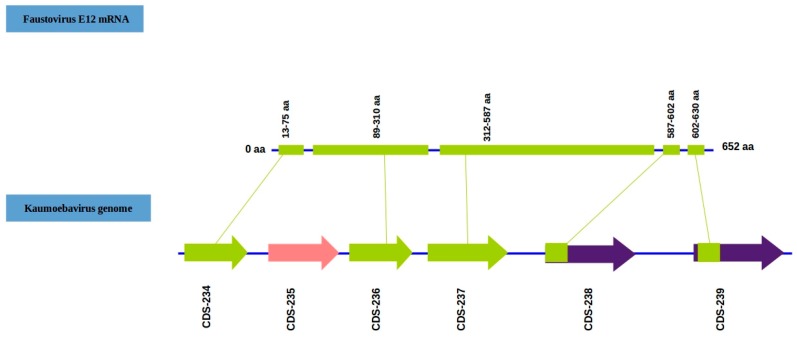
Overview of the genomic region in Kaumoebavirus that encodes the major capsid protein and the corresponding match in the Faustovirus E12 mRNA. The sequences coding for the major capsid protein are shown in green. A conserved nuclease found in the capsid region of both Faustoviruses and Kaumoebavirus is colored pink. Regions of Kaumoebavirus annotated as ORFs having a partial similarity with Faustovirus E12 mRNA are colored purple; only the parts with high similarity to Faustovirus E12 mRNA are colored green.

In addition, the phylogeny of several conserved genes shows the same result (Supplementary Materials Figures S3–S6). From an evolutionary perspective, Kaumoebavirus appears as an intermediate, close to Faustoviruses and *Asfarviridae*. The Kaumoebavirus genomic coding sequence undergoes splicing, just as Faustovirus. Indeed, the capsid protein-encoding gene of Faustovirus spans a 5 kbp region (180,327 bp–185,966 bp), including six open reading frames (ORFs), five of which are exons separated by putative introns (Figure 5).

## 4. Discussion

Co-culture with *V. vermiformis* enabled the isolation of a new giant DNA virus from a sewage water sample obtained in the southern part of Jeddah. Although this virus, named Kaumoebavirus, replicates in *V. vermiformis*, its genome organization and gene content indicate that it does not belong to the recently described Faustoviruses [17,18]. The TEM images of Kaumoebavirus show penetration through phagocytosis, as described in previous studies for other giant viruses and newly formed virions within the virus factory (contrary to *Acanthamoeba polyphaga mimivirus* (APMV), for which the newly formed virions are observed only at the periphery of the virus factory) [1,6,17]. In comparison with Faustovirus, phagocytosis and internalization of the individual Kaumoebavirus viral particles by the amoeba occurred between two and three hours, the same as in the case of Faustovirus [17]. This is late, as compared to other giant viruses such as APMV, but likely due to the behavior of its amoeba host *V. vermiformis*. However, the eclipse phase of this virus occurred between four and five hours PI, which is shorter than for Faustovirus (around six hours PI). Like Faustovirus, the replication cycle of Kaumoebavirus lasts between 16 and 20 h [17]. 

The phylogenetic study clusters Kaumoebavirus as a distant relative of Faustoviruses and *Asfarviridae*. This suggests that all these viruses represent an independent cluster within the proposed *Megavirales* order, composed of the family *Asfarviridae* and two other putative families, “*Faustoviridae*”, and Kaumoebavirus as a first isolate of a possible new family. Indeed, comparative genomics shows that the size of the core genome decreased dramatically when the Kaumoebavirus genome was included in the analysis, compared to the sizes estimated for the Faustoviruses (nine genomes) and the *Asfarviridae* (five genomes) genomes taken separately (Supplementary Materials Figure S7). However, its phylogenetic position is not yet clear. It is currently not possible to decide whether it has an ancestral or posterior relationship with *Asfarviridae* and Faustoviruses. The discovery of other viruses in this cluster could provide a more robust and intact phylogeny. Our characterization of Kaumoebavirus shows a dsDNA circular genome of 350 kb. Kaumoebavirus adds a potential new giant virus family with its 0.35 Mb genome. The Kaumoebavirus genome is smaller than that of Pandoraviruses, *Pithovirus sibericum, Mollivirus sibericum*, Faustoviruses and Mimiviruses. The range of GC content of the NCLDVs family members ranges from 17% (Entomopoxvirinae) to 64% (Parapoxvirus) [30]. Kaumoebavirus has a 43.7% GC content and the *Marseilleviridae* GC content’s value is the closest one to this virus. In addition to the phylogenetic link between Faustovirus and Kaumoebavirus, the latter seems to present the same dispersed structure of the capsid [31]. Indeed, the Kaumoebavirus capsid protein gene is split into five exons. The majority of Kaumoebavirus-encoded proteins (73%) do not have homologs, even in metagenomics databases such as environmental protein sequences database (env_nr). Of the Kaumoebavirus proteins presenting homology in the databases, they were more similar to eukaryotes (25%) than to bacteria proteins (15%), which is not surprising for a virus that infects eukaryotes.

## Supplementary Materials

The following are available online at www.mdpi.com/1999-4915/8/11/278/s1, Figure S1. No large-scale repeated regions were observed in the Kaumoebavirus genome. A: Pairwise sequence alignment of the Kaumoebavirus against itself (dot plot). B: Pairwise sequence alignment of the concatenated peptide sequences of Kaumoebavirus proteins. Figure S2. Concatenated proteins pairwise sequence alignment (dot plot) of Kaumoebavirus against itself and the fully sequenced Faustovirus E9, Faustovirus Liban and African swine fever virus strain E75. The Kaumoebavirus retains no observed collinearity with the other viruses. Figure S3. Phylogenetic reconstruction performed for Kaumoebavirus and other *Megavirales* members. Phylogenetic reconstruction was performed using the maximum likelihood method and was based on A32-like packaging ATPase. Figure S4. Phylogenetic reconstruction performed for Kaumoebavirus and other *Megavirales* members. Phylogenetic reconstruction was performed using the maximum likelihood method and based on D5-like helicase. Figure S5. Phylogenetic reconstruction performed for Kaumoebavirus and other *Megavirales* members. Phylogenetic reconstruction was performed using the maximum likelihood method and based on DNA topoisomerase. Figure S6. Phylogenetic reconstruction performed for Kaumoebavirus and other *Megavirales* members. Phylogenetic reconstruction was performed using the maximum likelihood method and based on the transcription termination factor. Figure S7. Comparative genomics of Kaumoebavirus and representatives of Faustoviruses and *Asfarviridae* family members.
