# Supplementary material for: Kaumoebavirus, a New Virus That Clusters with Faustoviruses and Asfarviridae
Source: Viruses. 2016 Oct 28;8(11):278. doi: 10.3390/v8110278 (PMC5127008; doi:10.3390/v8110278)
Supplement: Supplementary file 1 [file viruses-08-00278-s001.docx]

Supplementary Materials: Kaumoebavirus, a New Virus That Clusters with Faustoviruses and *Asfarviridae*

Leena H. Bajrai, Samia Benamar, Esam I. Azhar, Catherine Robert, Anthony Levasseur,
Didier Raoult and Bernard La Scola


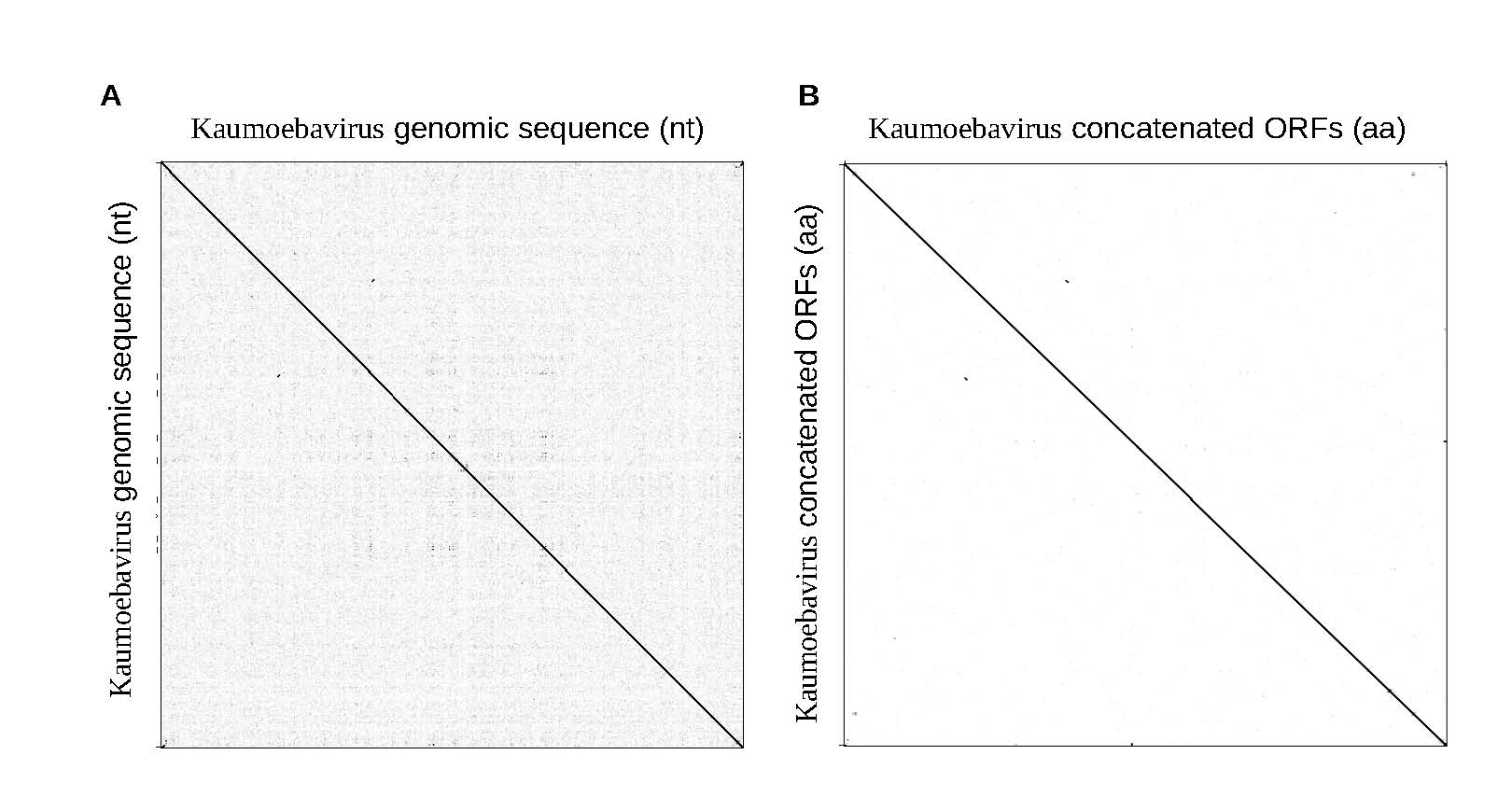


**Figure S1.** No large-scale repeated regions were observed in the Kaumoebavirus genome. (**A**) Pairwise sequence alignment of the Kaumoebavirus against itself (dot plot); (**B**) Pairwise sequence alignment of the concatenated peptide sequences of Kaumoebavirus proteins.


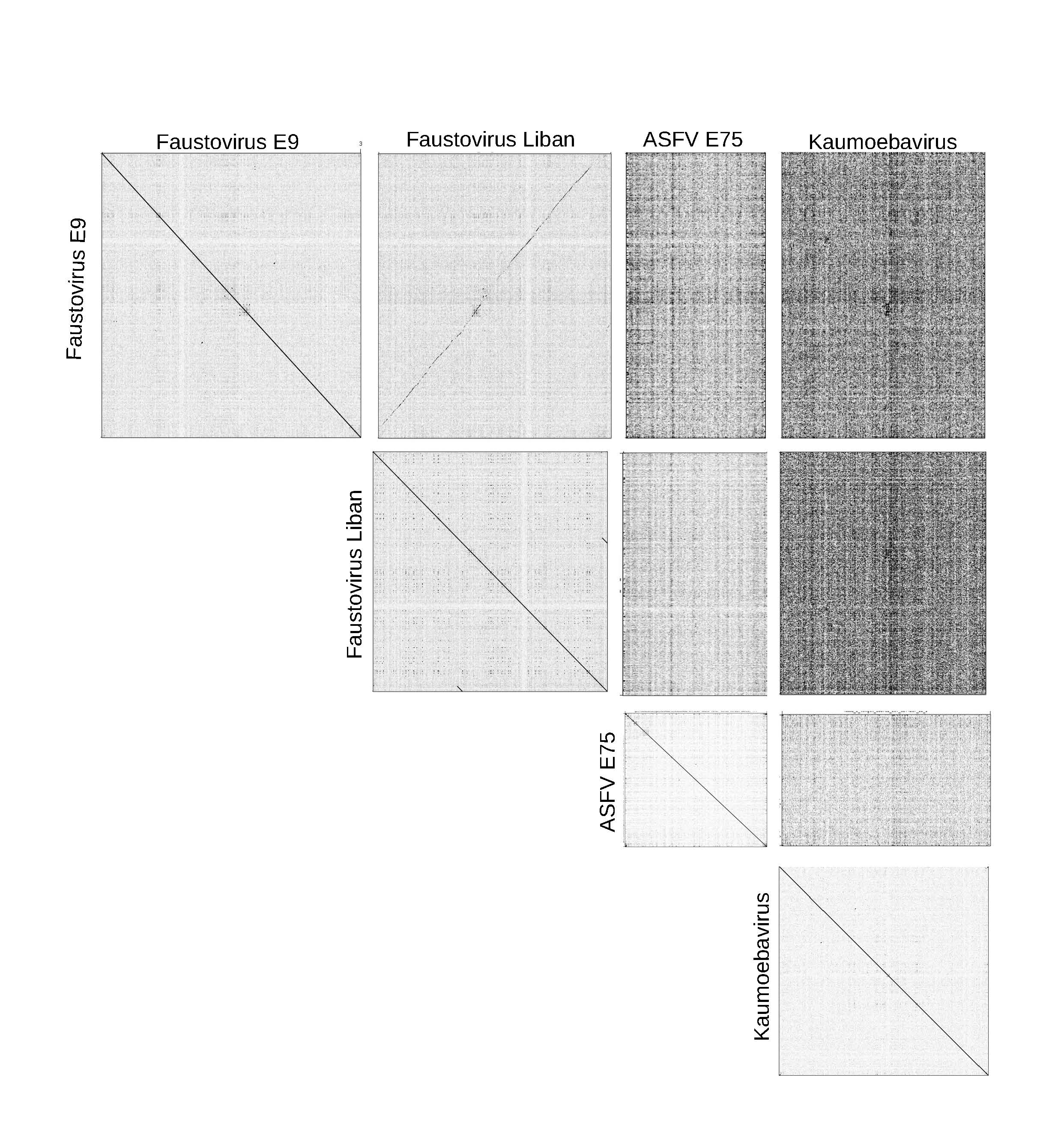


**Figure S2.** Concatenated proteins pairwise sequence alignment (dot plot) of Kaumoebavirus against itself and the fully sequenced Faustovirus E9, Faustovirus Liban and African swine fever virus strain E75. The Kaumoebavirus retains no observed collinearity with the other viruses.


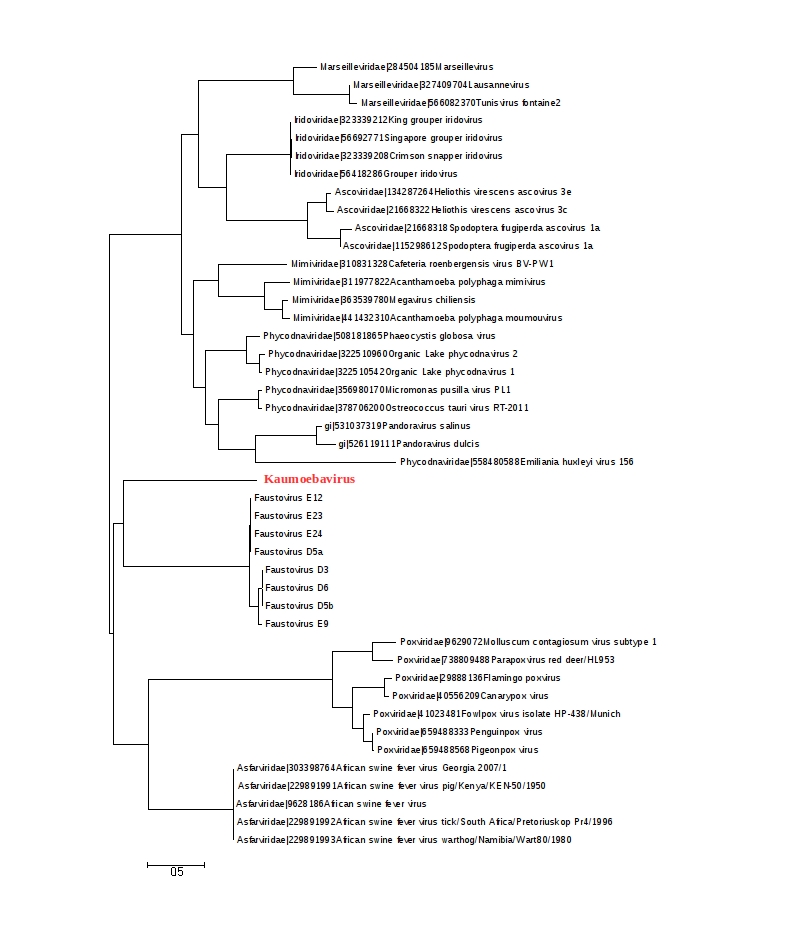


**Figure S3.** Phylogenetic reconstruction performed for Kaumoebavirus and other *Megavirales* members. Phylogenetic reconstruction was performed using the maximum likelihood method and was based on A32-like packaging ATPase.


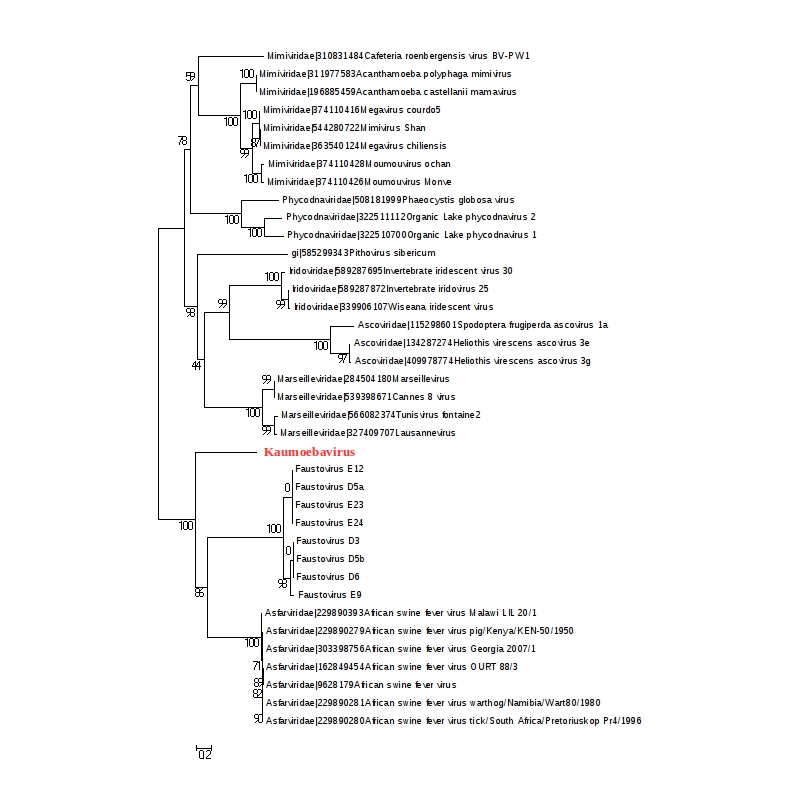


**Figure S4.** Phylogenetic reconstruction performed for Kaumoebavirus and other *Megavirales* members. Phylogenetic reconstruction was performed using the maximum likelihood method and based on D5-like helicase.


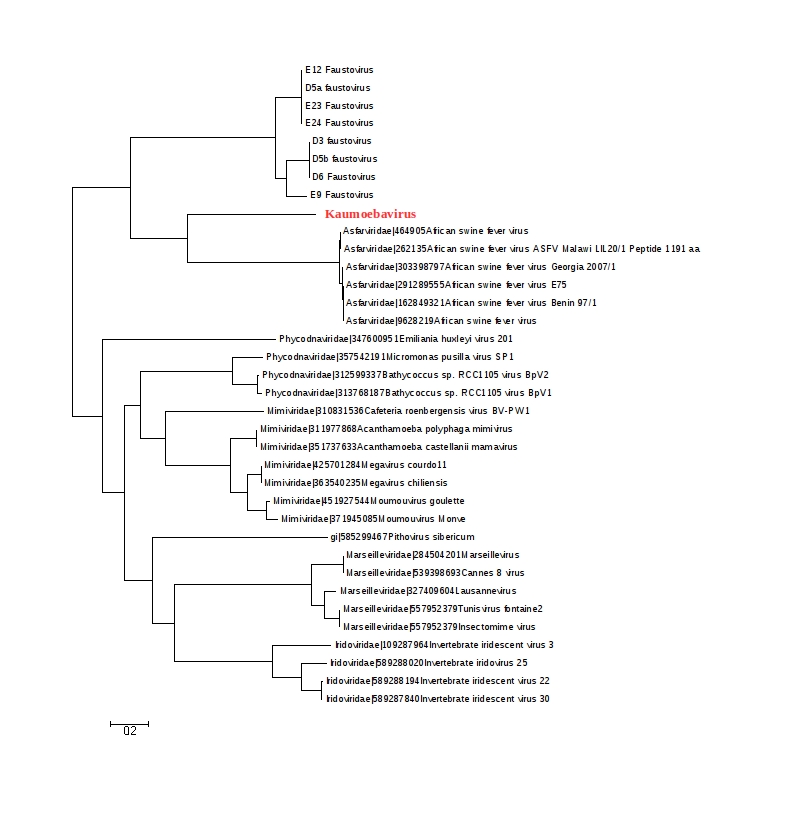


**Figure S5.** Phylogenetic reconstruction performed for Kaumoebavirus and other *Megavirales* members. Phylogenetic reconstruction was performed using the maximum likelihood method and based on DNA topoisomerase.


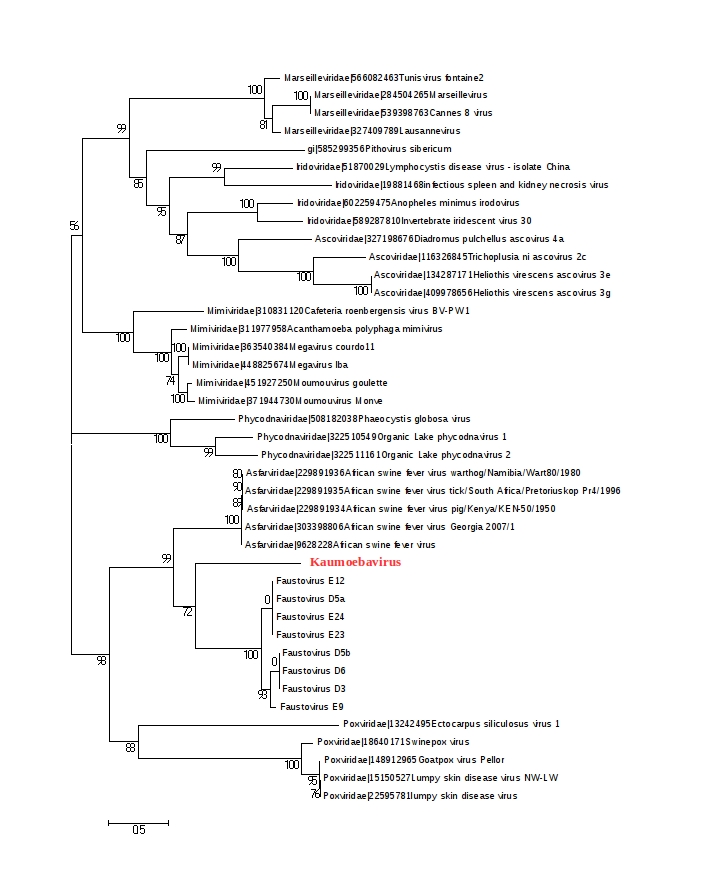


**Figure S6.** Phylogenetic reconstruction performed for Kaumoebavirus and other *Megavirales* members. Phylogenetic reconstruction was performed using the maximum likelihood method and based on the transcription termination factor.


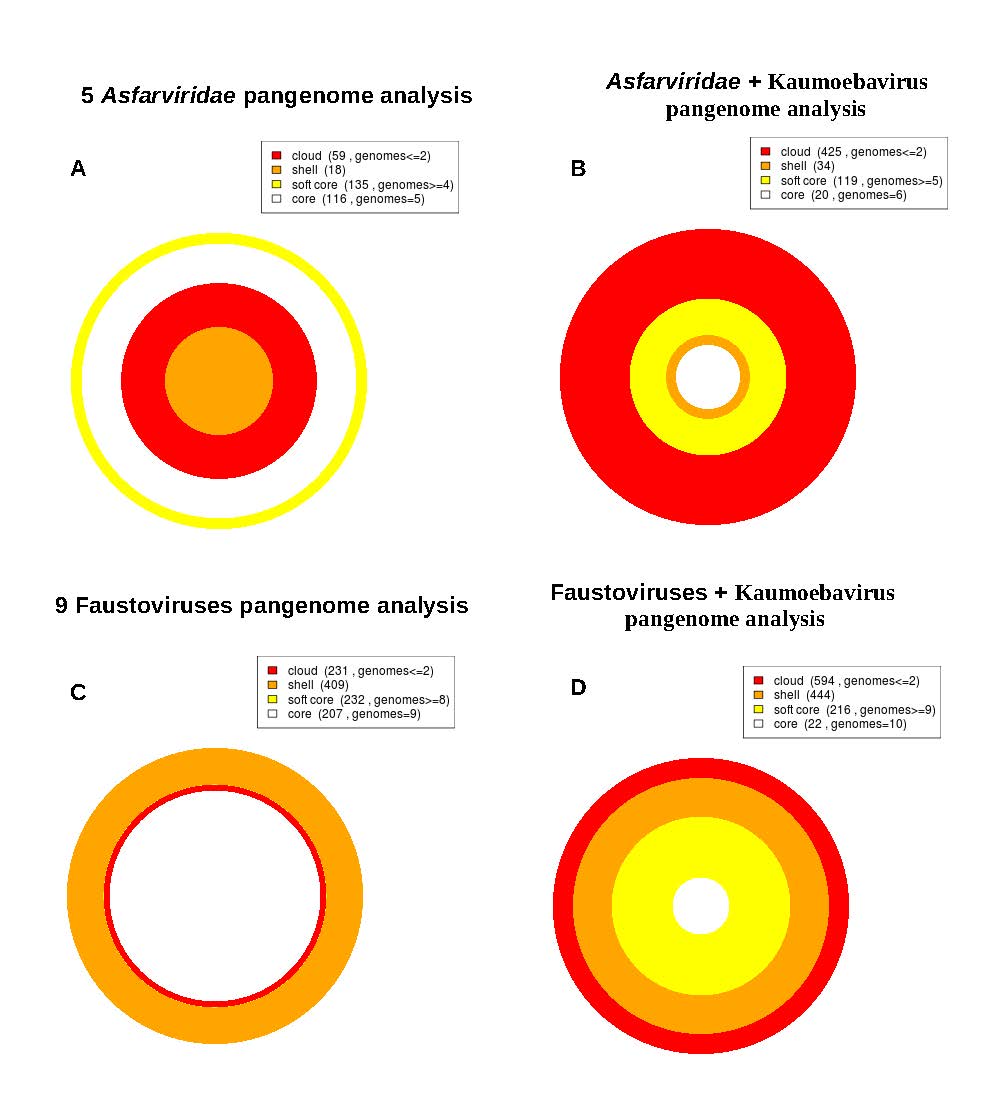


**Figure S7.** Comparative genomics of Kaumoebavirus and representatives of Faustoviruses and *Asfarviridae* family members.
